# Characterisation of Nitric Oxide Synthase in Three Cnidarian-Dinoflagellate Symbioses

**DOI:** 10.1371/journal.pone.0010379

**Published:** 2010-04-28

**Authors:** Helena Safavi-Hemami, Neil D. Young, Jason Doyle, Lyndon Llewellyn, Anke Klueter

**Affiliations:** 1 Department of Biochemistry and Molecular Biology, University of Melbourne, Melbourne, Victoria, Australia; 2 Faculty of Veterinary Science, University of Melbourne, Melbourne, Victoria, Australia; 3 Australian Institute of Marine Science, Townsville, Queensland, Australia; 4 School of Marine Sciences, University of Maine, Orono, Maine, United States of America; Louisiana State University, United States of America

## Abstract

**Background:**

Nitric oxide synthase (NOS) is an enzyme catalysing the conversion of L-arginine to L-citrulline and nitric oxide (NO), the latter being an essential messenger molecule for a range of biological processes. Whilst its role in higher vertebrates is well understood little is known about the role of this enzyme in early metazoan groups. For instance, NOS-mediated signalling has been associated with Cnidaria-algal symbioses, however controversy remains about the contribution of enzyme activities by the individual partners of these mutualistic relationships.

**Methodology/Principal Findings:**

Using a modified citrulline assay we successfully measured NOS activity in three cnidarian-algal symbioses: the sea anemone *Aiptasia pallida*, the hard coral *Acropora millepora*, and the soft coral *Lobophytum pauciflorum*, so demonstrating a wide distribution of this enzyme in the phylum Cnidaria. Further biochemical (citrulline assay) and histochemical (NADPH-diaphorase) investigations of NOS in the host tissue of *L. pauciflorum* revealed the cytosolic and calcium dependent nature of this enzyme and its in situ localisation within the coral's gastrodermal tissue, the innermost layer of the body wall bearing the symbiotic algae. Interestingly, enzyme activity could not be detected in symbionts freshly isolated from the cnidarians, or in cultured algal symbionts.

**Conclusions/Significance:**

These results suggest that NOS-mediated NO release may be host-derived, a finding that has the potential to further refine our understanding of signalling events in cnidarian-algal symbioses.

## Introduction

During the last two decades the importance of the enzyme nitric oxide synthase (NOS) has been demonstrated across a range of taxa including higher vertebrates [1] invertebrates [2], [3] and plants [4]. Across these diverse taxonomic groups, the functional role of NOS is often difficult to define due to the ubiquitous role of its product, nitric oxide (NO). In mammals, NO functions as an important signal transduction molecule and plays a key role in the immune [5], vascular [6] and nervous systems [7]. Studies in invertebrate systems have shown that NOS plays a role in mediating signalling processes, including organogenesis in *Drosophila melanogaster* [8] and the feeding response of the hydroid *Hydra vulgaris* [2], a member of the phylum Cnidaria. Many cnidarian species exhibit a close symbiotic relationship with unicellular dinoflagellates of the genus *Symbiodinium*. These algal symbionts provide photosynthetically derived compounds, which in many cases are essential for the host's survival [9]. Formation of NO by a NOS-like enzyme has been demonstrated for the host partner of cnidarian-algae symbioses [10], [11], however, controversy exists about the presence of this enzyme in the algal symbionts [11], [12], [13].

Recent studies have demonstrated low basal NOS activities within the symbiotic dinoflagellates from stressed and non-stressed animals [10], [13] with higher enzyme activities in algal cells experiencing heat stress [13]. It was suggested that algal NOS may influence physiological responses of the host [10] and potentially play a regulatory role in the animal's stress response system [13]. Perez and Weis (2006) explored the role of NOS in cnidarian bleaching and suggested that NOS activities measured in symbiotic algae may have been a result of host-NOS contamination [11]. Whilst a lot of knowledge exists for NOS signalling pathways and NOS-encoding genes in animals, for plants this knowledge is still sparse and in need of further investigation. Here we examine NOS activity in three distinct cnidarian species and their symbiotic dinoflagellates and further investigate the enzyme in the soft coral *L. pauciflorum*.

## Results

NOS activity was highest in *A. pallida*, lowest in *A. millepora* and intermediate in *L. pauciflorum* (Table 1). Using the modified NOS assay and protein concentrations ranging from 0.16–0.26 mg protein mL^−1^, NOS activity could not be detected in symbiotic dinoflagellates freshly isolated from any of the three cnidarian species, nor in cultured symbionts. Given that NOS activity was yet to be determined for members of the order Alcyonacea, we proceeded with further characterisation of NOS in *L. pauciflorum*.

**Table 1:** NOS activity in the host tissues of three cnidarian species.

Species	NOS activity (pmol citrulline *x* mg protein^−1^ *×*30 min^−1^)
*Acropora millepora*	25.2* (±10)
*Lobophytum pauciflorum*	376.4* (±8)
*Aiptasia pallida*	8127.5* (±76)

Asterisks depict significant difference in NOS activity (Tukey-Kramer Test, α = 0.05). Values are means of 3 individual experiments (± SE).

**Figure 1:**
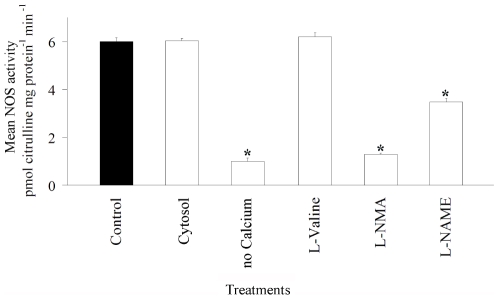
NOS activity in the host tissue of *Lobophytum pauciflorum*. Cellular localisation, calcium dependency and effects of inhibitors. NOS activity (pmol citrulline mg protein^−1^ min^−1^) of total protein extracts (control) was compared to samples (1) containing cytosolic extracts only, (2) incubated without calcium in the incubation solution, pre-incubated with (3) the arginase inhibitor L-valine (1 mM), the NOS inhibitors (4) L-NMA (1 mM) and (5) L-NAME (1 mM). Asterisks indicate significant differences from control treatments (N = 3, ± SE, Tukey-Kramer Test, α = 0.05).

Measurements of NOS activity in cytosolic fractions and crude homogenates containing membranous material were similar (*p*<0.87; Figure 1), strongly indicating that NOS is localised in the cytosol. Enzyme activity decreased significantly when calcium was excluded from the incubation solution (*p*<0.01; Figure 1), implicating calcium as an essential co-factor of NOS in the host tissue of *L. pauciflorum*. A significant reduction in NOS activity occurred for samples pre-incubated with the NOS inhibitors L-NMA and L-NAME (*p*<0.01, Figure 1). In addition to NOS, the enzyme arginase is also known to convert L-arginine to L-citrulline [10]. To ensure that all activity measured by the citrulline assay was due to NOS, the effect of the arginase inhibitor L-valine was assessed. L-valine (1 mM) had no effect upon the conversion of L-arginine to L-citrulline (*p*<0.48; Figure 1) demonstrating the specificity of our assay.

**Figure 2:**
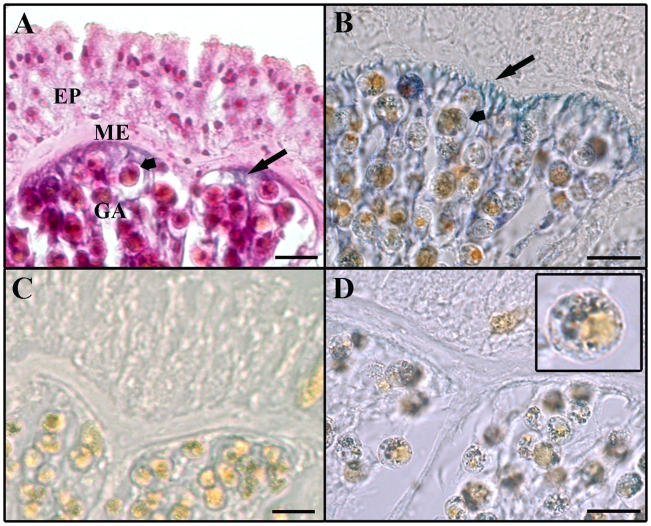
Cross-sections of *Lobophytum pauciflorum* tissue showing areas of NADPH-diaphorase staining. **A**: Section counterstained with H&E. **B**: NADPH-diaphorase staining evident in host tissue (long arrows) and in symbiotic dinoflagellates (short arrows). **C**: Negative control without staining; the NOS cofactor NADPH was omitted from the reaction. **D**: Negative control; the NOS inhibitors L-NMA and L-NAME were added to the staining reaction. NADPH-diaphorase staining is evident in dinoflagellates (insert) but not in host tissue. EP: epidermis, GA: gastrodermis, ME: mesoglea. Scale bars: 20 µm.

NBT staining was evident in the soft coral's gastrodermal tissue with the highest intensity in areas adjacent to the mesoglea (Figure 2B). Staining also occurred within some algal cells. The NOS-mediated conversion of L-arginine is NADPH-dependent. NBT staining was not observed in the negative control reaction omitting NADPH from the incubation solution (Figure 2 C, control without staining). In the 2^nd^ negative control reaction containing NOS inhibitors staining was evident within algal cells, but not within host tissues (Figure 2 D, insert).

## Discussion

Using a modified citrulline assay we demonstrated NOS activity in three phylogenetically distinct cnidarian species with significant differences in enzyme activities. Consistent with the findings of Perez and Weis (2006) no activity was observed in symbiotic dinoflagellates freshly isolated from the three cnidarian species nor in cultured algal cells. The alcyonacean coral *L. pauciflorum* was further investigated, as NOS had not yet been characterised in soft corals, keystone species in coral reef and other ecosystems. Further examination revealed that the enzyme was predominantly localised in the host's cytosol, a finding previously reported for *A. pallida* [14]. NOS activity could not be detected in the mucus, sensory and nerve cell-rich epidermal layer, therefore NOS is unlikely to be involved in the coral's sensory or nervous system as demonstrated for some asymbiotic cnidarians such as the jellyfish *Aglantha digitale* [3] and the hydroid *Hydra viridis* [2]. Interestingly, the gastrodermis of *L. pauciflorum* appeared to be the central tissue of NOS activity. The gastrodermis is not only responsible for digestion and reproduction but also bears the symbiotic algal cells [15]. Nitric oxide is a highly reactive gaseous molecule able to diffuse across cell membranes. Given the localisation of NOS activity in the coral's gastroderm, NOS-mediated release of NO is likely to affect the symbiotic algae. Furthermore NOS activity appeared to be calcium dependent concurrent with results obtained for the sponges *Axinella polypoides* and *Petrosia ficiformis* [16], the most primitive group of Metazoa. In sponges, animals lacking a nervous system, NOS activity was suggested to be involved in cellular signalling events, in particular during heat stress events [16]. Similarly, recent studies have implemented NOS in the heat-stress response of *A. pallida* [11] and the hard coral *Madracis mirabilis* [13]. However, uncertainty remains about the contribution of NOS-mediated NO released by the individual partners to the symbiotic relationship. While low basal NOS activities have been reported for symbiotic algae with increasing enzyme activities in algal cells experiencing heat stress [13], subsequent studies using fluorescent detection methods attributed this to host contamination [11]. Using a similar approach to Perez and Weis (2006), NO production was shown to increase in cultures of *Symbiodinium microadriaticum* exposed to heat stress [12]. The authors attributed NO release to an algal NOS-like enzyme but also acknowledged a potential contribution of other enzymes such as nitrate reductases [17]. Current methods of monitoring NOS activity in symbiotic dinoflagellates clearly produce ambiguous results and alternative approaches are required to resolve the putative role of this enzyme in algae. Whilst NADPH-diaphorase staining proved to be a reliable technique to identify NOS activity in the host tissue of *L. pauciflorum*, the lack of knowledge on potential algal NOS inhibitors does not allow for accurate testing of algal NOS using this method as yet. Generally, the citrulline assay used here is a very sensitive and reliable method for the determination of NOS activity, however it is not suitable to specifically localise NOS activity.

By using a combination of complementary techniques we demonstrate NOS activity in *L. pauciflorum* to be calcium-dependent and host-derived. Due to its localisation in the coral's gastroderm we suggest a possible role in host-algae interactions. However, this requires further investigations, and techniques to characterise and measure NOS in both partners need to be carefully evaluated.

## Materials and Methods

The Great Barrier Reef Marine Park Authority provided permits for this work (G02/4011.1).

### Study species and location

Specimens of *Lobophytum pauciflorum* and *Acropora millepora* were collected from Bay Rock (19°7′S/146°45′E) and Magnetic Island (19°10′S/146°51′E), Townsville, Australia. Within 2 hours of collection corals were transferred to a 500-litre tank with aeration and flow-through seawater (∼2 L min^−1^, 26°C). Corals were acclimatised for 10 days prior to sampling. Specimens of *Aiptasia pallida* were obtained from cultures maintained at the Australian Institute of Marine Science and held under similar conditions to those described for corals.

### Preparation of tissue homogenates and algal cell isolates

Entire *A. pallida* (4 cm in diameter) and pieces (2×2 cm) of *L. pauciflorum* were homogenized for 20 s in 5 mL of ice-cold homogenization buffer (HB containing 50 mM Tris, 1 mM EDTA, protease inhibitor cocktail (Complete, Roche), pH 7.4, using a Teflon/glass Potter homogenizer. *Acropora millepora* tissue was removed from the skeleton by air brushing and homogenized for 20 s in ice-cold HB using probe homogenisation (PRO Scientific, PRO250, Australia). Symbiotic dinoflagellates from each of the three cnidarian species were isolated by subsequent centrifugation (3,500 rpm, 5 min, 4°C) of the tissue homogenate and filtration through a 20 µm nylon filter (Millipore, Australia) [18]. Host proteins were isolated from the remaining tissue homogenates by centrifugation (14,000 rpm, 10 min, 4°C) and collection of the supernatant. Algal proteins were isolated from the purified dinoflagellate cells *via* sonication (60 s at 40 Hz on CaCl_2_ ice (1 M)). Protein concentrations of both host and algae were determined using the Bradford method [19] (Bradford Reagent B 6919, Sigma, I1, USA).

### Culture of symbiotic dinoflagellates

Freshly isolated symbiotic dinoflagellates were further purified by centrifugation (3,500 rpm, 5 min, room temperature (RT)) and cultured *ex vivo* in Erd-Schreiber media [20] with the addition of GeO_2_ (2.5 mg L^−1^), G-Penicillin (80 mg L^−1^), Streptomycin (80 mg L^−1^), Amphotericin (40 mg L^−1^), Thiamine HCl (0.4 mg L^−1^), Biotin (2 µg L^−1^) and Vitamin B_12_ (2 µg L^−1^). Cultures were maintained at constant conditions (25°C, 12 h/12 h light/dark cycle and an irradiance of 100–140 µmol quanta m^−2^ s^−1^) for a minimum of 4 weeks prior to sampling.

### Measurement of NOS activity

The citrulline assay described by Bredt and Snyder (1989) [21] was modified for a 96-well microplate to allow for higher sample throughput. For each reaction, a 10 µL aliquot of protein containing 4.4 µg total protein was added to 40 µL of reaction buffer (final concentrations of: 0.6 µM CaCl_2_, 1 mM NADPH, 6 µM BH_4_, 4 µM FAD, 4 µM FMN, 170 units/mL calmodulin, 1 mM DTT; for host samples: 15 µM L-arginine (including 0.15 µM L-[2,3,4,5-^3^H] arginine monohydrochloride ([^3^H]-arginine,1 mCi/ml, 57 Ci/mmol, Amersham Biosciences, UK)) in 50 mM Tris-HCl pH 7.4); for algal samples: 35 µM L-arginine (including 0.35 µM [^3^H]-arginine). Samples were incubated at 25°C for 30 min. The reaction was stopped by adding 200 µL ice-cold stop buffer (50 mM HEPES, 5 mM EDTA, pH 5.5). A 150 µL aliquot was transferred to a clean microplate and mixed with 100 µL equilibrated ion-exchange resin (Dowex AG-50W (Na^+^ form), equilibrated with 50 mM HEPES, pH 6.0, Biorad, Australia). The plate was sealed and shaken gently for 25 min and centrifuged (2 min, 1000×*g* at RT). A 70 µL aliquot of supernatant was mixed with 200 µL scintillation liquid (Optiphase supermix, Wallac, Finland) in a 96-well plate, sealed and shaken gently for 4 hrs before the activity of radio-labelled product was measured. For negative controls protein extracts were boiled for 15 min at 100°C prior to NOS measurements. Positive controls contained recombinant rat neuronal NOS (Sigma, I1, USA).

### Characterisation of NOS in the host tissue of *Lobophytum pauciflorum*

To determine the subcellular localisation of NOS, a sample containing only cytosolic proteins of the crude homogenate was analysed. Cytosolic proteins were prepared by ultracentrifugation (100,000 *g* for 60 min, 4°C). Calcium dependency of host NOS was investigated by incubating total protein extracts with both standard reaction mixtures (50 mM Tris-HCl, pH 7.4) and reaction mixtures from which CaCl_2_ was omitted. The effects of the NOS inhibitors, N*_ω_*-Nitro-L-arginine (L-NMA) and *N_ω_*-Nitro-L-arginine methyl ester hydrochloride (L-NAME), as well as the arginase inhibitor L-valine were tested. NOS preparations were pre-incubated for 10 min in 1 mM of each inhibitor prior to measurements of enzyme activity as described above.

### NADPH-diaphorase histochemistry for *Lobophytum pauciflorum*

The body wall of coral polyps consists of an outer epidermal and inner gastrodermal layer separated by a non-cellular matrix called the mesoglea [15]. To localise NOS activity within these structures, coral tissue was stained with nitroblue tetrazolium (NBT) utilizing the NADPH-diaphorase (NADPH-d) technique [3]. Corals were anaesthetised with 0.3 M MgCl_2_ for 10 min followed by a thorough wash with filtered seawater (FSW, UV sterilized, 0.2 µm). Explants were taken from the colony's centre and cut into transverse sections. Cross sections were prepared according to Moroz *et al.* (2004). For pre-fixation cross sections were immersed in FSW containing 4% w/v paraformaldehyde and 0.1% w/v glutaraldehyde and incubated at RT. For pre-fixation, two different times were tested, 15 and 30 min. Following pre-fixation, sections were washed 3 x for 10 min in 0.5 M Tris-HCl, pH 8.0. Washed sections were incubated in fresh incubation solution, containing 0.5 mM NBT, 0.1 mM dicumarol, 1 mM NADPH, 0.25% Triton X-100 in 0.5 M Tris-HCl, pH 8.0. Samples were incubated in the dark for 30 min at RT. The incubation was stopped by washing the sections 3 x for 10 min in 0.5 M Tris-HCl pH 8.0. For post-fixation, sections were treated for 30 min in 4% paraformaldehyde/methanol to remove non-specific staining followed by dehydration and wax embedding using an automated tissue-processor (Shandon Hypercenter XP, USA). Embedded samples were cut using a microtome (10 µm sections; Microm Heidelberg HM330, Germany), cleared with xylene and mounted in fast drying mounting medium (United Biosciences, Australia). To determine the integrity of coral tissue selected sections were counterstained with Haematoxylin & Eosin following routine histological procedures. Two negative controls were performed: 1. NADPH was omitted from the incubation solution, 2. Staining not derived from NOS activity was visualised by adding 1 mM of the NOS inhibitors L-NMA and L-NAME to the incubation solution.
